# Mechanisms and significance of therapy-induced and spontaneous senescence of cancer cells

**DOI:** 10.1007/s00018-019-03261-8

**Published:** 2019-08-14

**Authors:** Justyna Mikuła-Pietrasik, Arkadiusz Niklas, Paweł Uruski, Andrzej Tykarski, Krzysztof Książek

**Affiliations:** Department of Hypertensiology, Angiology and Internal Medicine, Poznan University of Medical Sciences, Długa 1/2 Street, 61-848 Poznan, Poland

**Keywords:** Cancer biology, Cellular senescence, Spontaneous senescence, Therapy-induced senescence

## Abstract

In contrast to the well-recognized replicative and stress-induced premature senescence of normal somatic cells, mechanisms and clinical implications of senescence of cancer cells are still elusive and uncertain from patient-oriented perspective. Moreover, recent years provided multiple pieces of evidence that cancer cells may undergo senescence not only in response to chemotherapy or ionizing radiation (the so-called therapy-induced senescence) but also spontaneously, without any external insults. Since the molecular nature of the latter process is poorly recognized, the significance of spontaneously senescent cancer cells for tumor progression, therapy effectiveness, and patient survival is purely speculative. In this review, we summarize the most up-to-date research regarding therapy-induced and spontaneous senescence of cancer cells, by delineating the most important discoveries regarding the occurrence of these phenomena in vivo and in vitro. This review provides data collected from studies on various cancer cell models, and the narration is presented from the broader perspective of the most critical findings regarding the senescence of normal somatic cells.

## Introduction

Paraphrasing Heraclitus’ philosophy *panta rhei* (“everything flows”), the concept that everything is transient and temporary, a current biogerontologist could summarize the knowledge accumulating in the aging field over the past century with a statement that “everything is getting old”. Since the early 20th century, a group of researchers believed that cells might be, in their nature, immortal [1]. These ideas were crushed when Leonard Hayflick and Paul Moorhead discovered that human somatic cells (precisely: lung fibroblasts) might achieve, in vitro, only a finite number of population doublings and before becoming old (or *senescent*, according to an adequate terminology) [2, 3]. The next few decades resulted in an extended list of cells whose Hayflick limit and mechanism of senescence were revealed. The list now includes, among others, fibroblasts, epithelial cells, endothelial cells, mesothelial cells, and mesenchymal stem cells. The previously popular paradigm that cancer cells do not become senescent, and thus are immortal [4], dramatically changed when their senescence in response to chemo- and radiotherapy was documented [5]. The last fortress for those held onto the notion that cell types which need to be immortal, for their own identity, were cancer cells not experiencing any external insult. Unfortunately, these arguments failed when the spontaneous senescence of cancer cells was described [6]. This review is devoted to detailing the molecular and mechanistic aspects of the spontaneous senescence of cancer cells which, in comparison to other types of this process, is still poorly explored, full of puzzles, and underestimated from a clinical perspective.

## Cellular senescence

Alexis Carrel’s theory about cell (and possibly human) immortality [1, 7] collapsed when Hayflick and Moorhead failed to reproduce his observations. Carrel’s findings were then challenged by showing the inability of normal somatic cells to divide beyond some fixed and predetermined number of replications, and an apparent morphological deformation of those cells at late passages [2, 3]. Current perspectives on mechanisms and outcomes of cellular senescence, a process reported for the first time in the early 60s of twentieth century, gradually evolved in parallel with methodological advances, particularly in the area of cell and molecular biology. The fibroblast is the most common model of cellular senescence, with the vast majority of discoveries in this field stemming from studies on this cell type [8].

### Phenotype

A senescent cell degenerates and in contrast to young, proliferating, and also quiescent cells, senescent cells are usually much bigger, sometimes several times. There are probably several mechanisms underlying this senescence-associated cell enlargement. One of them is cellular hypertrophy in which a cell becomes bigger and bigger due to an accumulation of proteins [9]. It has been postulated that accumulation of proteins in senescent cells may be associated with decreased activity of proteasomal peptidases combined with increased levels of either oxidized or ubiquitinated proteins [10]. Senescent cells also lose monolayer integrity which may result from a downregulation of intercellular junctions [11, 12], display irregularities in shape (e.g., related to overproduction of vimentin, as it happens in senescent fibroblasts [13]), becoming multinucleated [14], vacuolarized [15], and developing altered mitochondria in terms of both morphology (e.g., increased mass [16]) and function [17].

Another significantly affected structure of senescent cells is their DNA, which gathers several forms of specific abnormalities, including products of base oxidation (e.g., 8-hydroxy-2′-deoxyguanosine [18]), persistent DNA damage foci (DNA segments with chromatin alterations reinforcing senescence; DNA-SCAR) [19], and senescence-associated heterochromatin foci (SAHF), responsible for silencing of proliferation-promoting genes [20]. Moreover, there is a rising conviction that senescence-related changes in DNA also occur at an epigenetic level [21].

Senescent cells exhibit the cytoplasmic activity of lysosomal β-galactosidase, which is detectable at pH 6.0, is called the senescence-associated β-galactosidase (SA-β-Gal) [22]. Although the presence of SA-β-Gal is probably the most widely used marker of senescent cells, a lot of criticism has appeared regarding the specificity of this enzyme for the senescence state [23, 24]. Currently, the list of senescence markers goes far beyond SA-β-Gal and includes the acquisition of senescence-associated secretory phenotype (SASP) [25], cell cycle inhibitors p16^Ink4a^ and p21^Cip1^ [26], and lipofuscin [27]. This multiplicity of senescence biomarkers, combined with a unique pattern of senescence in various cell types led to the current view in which senescent cells should be identified using not only SA-β-Gal but also using other common markers, such as histone γ-H2A.X, SAHF, and p16^Ink4a^. A confirmation that cells supposed to be senescent are negative for proliferating antigens, particularly Ki67, is also strongly advisable [28].

### Mechanisms

There is an agreement that senescence is a cell response to an extensive and irreparable DNA injury localized in various regions of the genome [29, 30]. Under certain conditions, however, the senescence may proceed without DNA damage [31]. Currently, two major types of cellular senescence are considered: replicative senescence and stress-induced premature senescence (SIPS). In general, these types of senescence are distinguished by the number of divisions at which the senescence occurs [32].

The classic pattern of senescence that defines cell fates upon reaching a certain, cell-specific number of divisions, is called *replicative senescence*. This kind of senescence is intuitively linked with changes in telomere length and structure (uncapping), and therefore, is also recognized as *telomere*-*dependent senescence*. Telomeric ends are particularly prone to DNA damage, mostly single-strand breaks (SSB) and double-strand breaks (DBS), due to defective DNA repair mechanisms [33]. Gradual decomposition of telomeres through shortening and/or uncapping induces the DNA damage response (DDR) pathway [34], which is responsible for a cell evacuating from the cell cycle. The DDR pathway starts from the recruitment of ataxia telangiectasia mutated (ATM) and ATM- and Rad3-Related (ATR) kinases to the sites with DSBs (a signature of damaged telomeres). This recruitment leads to the phosphorylation of hundreds of proteins, including histone H2A.X at Ser139 (γ-H2A.X), 53BP1, MDC1, NBS1, and kinases Chk1 and Chk2 [35]. These reactions result in the activation of p53 cell cycle checkpoint and an irreversible exit from the cell cycle at the stage of G_1_ or G_2_ phases [36]. The reinitiation of cell division is additionally blocked by the upregulation of another cell cycle inhibitor, p21^Cip1^, which is regulated by p53 at the transcriptional level and acts as its down-stream effector [37]. Although DDR is a universal reaction elicited by various forms of DNA injury, the accumulation of its activated elements in senescent cells justifies to treat it as the core signaling route for senescence [38, 39].

Research on cells other than fibroblasts has demonstrated that the telomere-dependent pattern of senescence is not the only mechanism of this process. Cells of epithelial nature, including keratinocytes, as well as cells of mesenchymal origin resembling in their morphology and function to epithelial cells (mesothelium), senesce with little to no change in telomere length [40, 41]. This version of senescence is termed SIPS, as its primary cause is inadequate culture conditions (“culture shock”) [42]. Cells undergoing SIPS seem to be more vulnerable to environmental insult than the cells undergoing replicative senescence, manifested by the larger magnitude of DNA damage, which additionally localizes predominantly in non-telomeric regions of the genome (*telomere*-*independent senescence*) [43]. Interestingly, the DDR is also activated in SIPS [44], albeit another cell-cycle blocker, p16^INK4a^, plays the critical role [45]. In the context of the executionary phase of senescence at the level of cell cycle, the involvement of certain inhibitors, like p21, p16, and p53 is highly cell-specific. For example, senescent fibroblasts that reached this state primarily in telomere-dependent fashion often display upregulated level of p16^Ink4a^ [46], whereas mesothelial cells which senesce prematurely without telomere shortening, are characterized by elevated p21^Cip1^ at senescence [43].

A special kind of the SIPS is the irreversible cessation of growth upon cell exposure to a variety of stressors or manipulations, including ultraviolet radiation [47], ionizing radiation [48], chemotherapeutics [49], and sub-lethal doses of oxidants [50]. In general, the SIPS occurs quickly, usually within a few rounds of replication and may have telomere-dependent [51] or telomere-independent characteristics [52]. The complexity of this phenomenon, that may proceed using various molecular pathways, has been shown by observations that under some specific conditions, even the DNA injury is not a binding element of the SIPS development [31].

Another kind of cellular senescence, known as *oncogene*-*induced senescence* (OIS), is associated with the activation of certain oncogenes. Although several oncogenes exist and play a role in the biology of normal and cancerous cells, the phenomenon of OIS has been described most extensively for their two families, that is *RAS* [53] and *RAF* [54]. Generally speaking, the activation of the oncogenes, usually through an ectopic expression of their activated forms, drives cells towards the development of the phenotype that characterizes cells undergoing replicative senescence and SIPS [55].

Oxidative stress is probably the best recognized, both intrinsic (mitochondrial) and environmental insult, whose effects lead to cellular senescence. In case of replicative senescence, oxidative stress is associated with compensatory biosynthesis of mitochondria in response to declined inner membrane potential (so-called retrograde signaling response) [56] and contributes to telomere shortening [57], next to the end-replication problem [58]. The retrograde signaling may also occur in cells that undergo SIPS [59]. There is also evidence that apart from oxidative stress resulting from the compensatory biogenesis of mitochondria, another mechanism of reactive oxygen species overproduction includes the increased activity of cytochrome c oxidase and NADH dehydrogenase, the enzymes that control the rate of electron flow through the electron transport chain [60].

When it comes to the SIPS, the exogenous oxidants trigger permanent cell growth cessation by the extensive DNA injury [61]. One of the best evidence for the causative role of oxidative stress in cellular senescence derives from experiments on fibroblasts which maintained under decreased oxygen pressure (hypoxia) displayed significantly improved replicative lifespan and delayed senescence [62]. A similar effect of hypoxia has also been observed in mesenchymal stem cells [63], osteoclasts [64], and human endothelial progenitor cells [65]. Hypoxia has also been found to prevent OIS, the effect of which was associated with the induction of hypoxia-inducible factor-1α (HIF-1α). Mechanistically, hypoxia downregulated ATM/ATR, Chk1 and Chk2 phosphorylation leading to attenuated DDR. Detailed analysis of HIF-1α activity revealed that it plays a role in targeting p53 and p21^Cip1^ and that its knock down leads to apoptosis, but not the restoration of senescence in *RAS*-expressing fibroblasts exposed to hypoxia [66].

Last but not least, it should also be mentioned that cellular senescence is not necessarily irreversible. It has been demonstrated that fibroblasts and mammary epithelial cells characterized by low level of p16^Ink4a^ at senescence may reinitiate dynamic growth upon p53 inactivation. This observation implies that senescence triggered by telomere dysfunction may be a reversible phenomenon, in the maintenance of which p53 plays a dominant role [67]. Earlier, similarly effective restoration of cell capacity to divide (replicative rejuvenation) was observed in cells in which telomerase was reactivated [68].

### Biological role

Cellular senescence is not only an in vitro phenomenon, or an artifact of cell culture, as was postulated by some authors [69]. The presence of senescent cells has been documented in various tissues, including in the skin [22], kidney [70], blood vessels [71], prostate [72], and peritoneal cavity [73], implying that they may play some role in these tissues in physiology and/or pathology. Answering the question about the role of senescent cells accumulating in vivo is far more difficult. Do senescent cells contribute to organismal aging? They likely do, which statement has recently been reinforced by findings showing that the ablation of p16^Ink4a^-positive senescent cells elongated median lifespan, attenuated tumorigenesis, and delayed onset of age-associated dysfunction of the heart, kidney, and fat in INK-ATTAC mice [74]. In addition, senescent cells are an integral part of the pathogenesis of age-associated pathologies, such as atherosclerosis [71], diabetes [75], benign prostate hyperplasia [72], osteoarthritis [76], cataracts [77], melanocytic naevi [78], cardiovascular disease [79], cognitive disorders [80], and cancer [81]. At the same time, there are reports which detail the beneficial role of senescent cells in wound healing [82] and embryogenesis [83], as well as an orchestrator of an immune system cell behavior [84] was described. Some authors even proposed the concept that senescent cells could be transiently delivered to an organism to engage their explicit secretory properties in regenerative purposes [85]. On the other hand, there is strong evidence that anti-aging drugs, so-called *senolytics*, are effective in eliminating senescent cells in vivo and in improving health span parameters in aged animals [86].

Despite the common agreement that cellular senescence acts as a tumor suppressor mechanism, particularly in young organisms [87], the last 2 decades have provided mounting evidence that senescent cells are causatively involved in cancer progression. Their contribution includes the formation of an immunosuppressive tissue microenvironment, e.g., through interleukin 6 (IL-6)-dependent stimulation of suppressive myeloid cells, and their ability to restrict anti-tumor T cell reactions [88]. Furthermore, senescent cells are characterized with the SASP, which refers to an overproduction of variety of cytokines (e.g., IL-1, IL-6, IL-7, IL-13), chemokines (CCL2, CXCL1, CXCL8, CXCL12), growth factors (heregulin, EGF, bFGF, IGF, VEGF, TGF-β1), and extracellular matrix (ECM) constituents and remodeling proteins (fibronectin, collagens, laminin, PAI-1, uPA, tPA, MMP-1, -3) known to participate in various steps of cancer cell progression [25]. Noteworthy, however, the composition of SASP may be different in various cell types, as their senescence, including transcriptional profile are highly heterogenous [89].

**Fig. 1:**
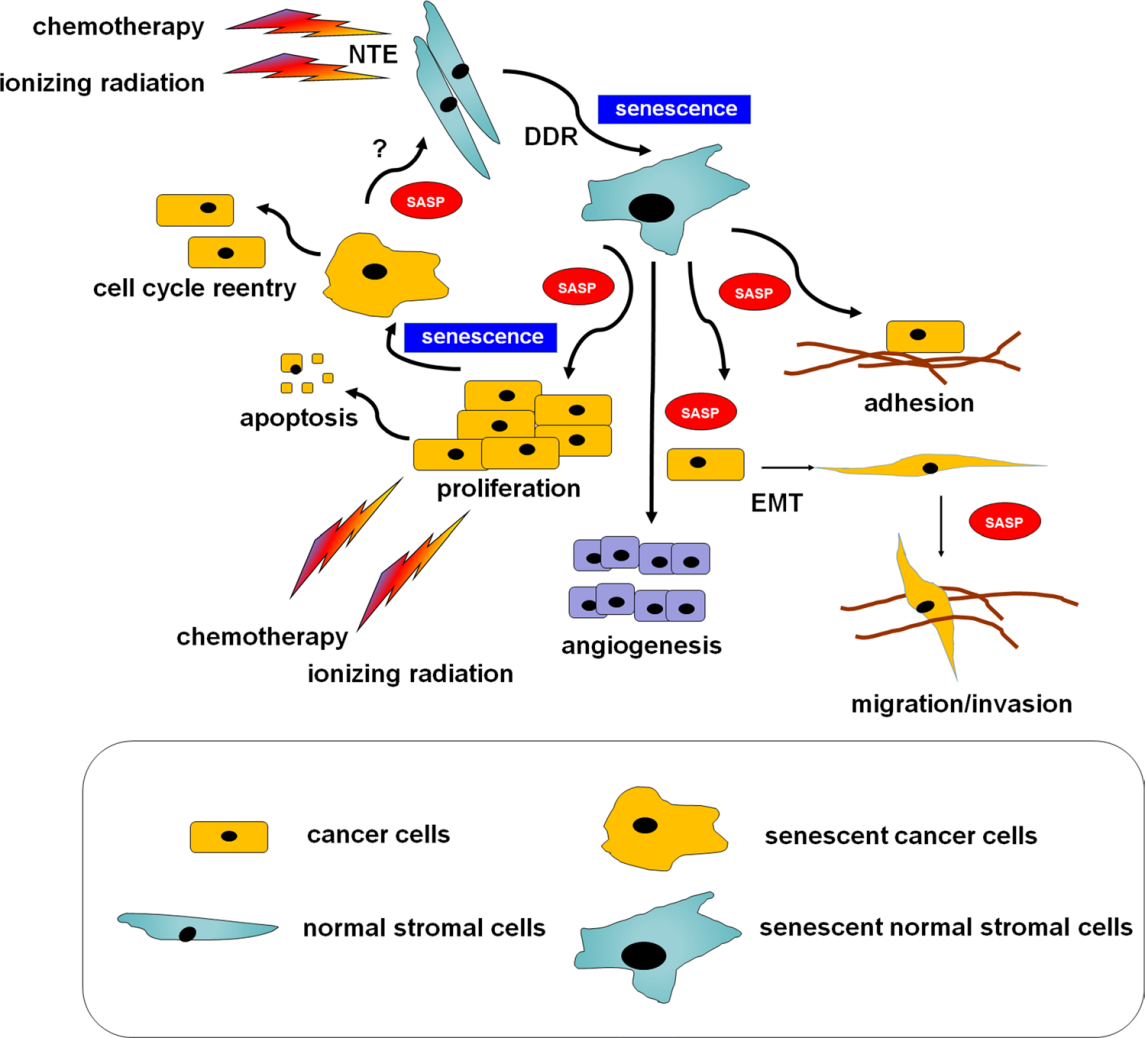
A hypothetical, cancer-modulating loop formed by normal stromal cells interacting with cancer cells undergoing therapy-induced senescence. According to the current knowledge, the phenomenon of cellular senescence may apply to either normal or cancer cells forming a tumor. In contrast to well-established cancer-promoting activity of senescent stromal cells, the outcomes of senescent cancer cells may be both pro- and anti-tumoral. It is still unknown whether senescent cancer cells may contribute to the induction of senescence in normal cells, e.g., via the SASP. *DDR* DNA damage response, *EMT* epithelial–mesenchymal transition, *NTE* radiation-induced non-targeted bystander, *SASP* senescence-associated secretory phenotype

Senescent cells have been found to support adhesion, proliferation, migration, and invasion of cancer cells, as well as stimulating cancer-supportive phenomena, like angiogenesis [90] and epithelial–mesenchymal transition (EMT) [91]. From the effector perspective, the SASP is controlled by p38 MAPK and NF-κB [92]. Recent studies suggest, however, that other pathways, including GATA4, mTOR, Jak2/Stat3, and the inflammasome also play a role [93]. Interestingly, the development of this phenotype may proceed in a mechanism engaging the DDR [94] or DDR-independently [95]. The pro-cancerous activity of senescent cells is not restricted to in vitro conditions. Experiments on laboratory animals showed that senescent cells stimulate the formation of breast [96], colorectal [97], pancreatic [98], and ovarian tumors [99] much more efficiently than their young counterparts (Fig. 1).

## Therapy-induced senescence of cancer cells

The paradigm that cancer cells are immortal was often linked with the statement that they proliferate indefinitely and avoid senescence due to active telomerase or alternative mechanisms of telomere lengthening [4]. For this reason, telomerase became a tempting target in experimental anti-cancer therapy [100]. The truth is, however, far more complex, which is evidenced by multiple observations that senescence may be triggered in cancer cells by their exposure to clinically relevant doses of ionizing radiation (radiotherapy) and chemotherapy [101]. This indicates that despite cancer cells needing to bypass senescence in the course of their immortalization, they preserved (or at least some of them preserved) intact molecular effector pathways leading to senescence, which may be activated under some, therapy-related circumstances.

### Radiation-induced senescence of cancer cells

Ionizing radiation (IR) is a common form of cancer therapy, based on the ability of the radiation to destroy DNA in cancer cells, leading to their death [102]. A body of evidence has accumulated showing that the IR induces cellular senescence in various cancer cell types, in a dose-dependent manner. In the non-small cell lung cancer (NSCLC) A549 cells, 2 Gy of radiation yielded ~ 20% of SA-β-Gal-positive cells, whereas 10 Gy generated the SA-β-Gal positivity in almost 80% of cells. This response is, however, also cell-type specific, as in the H460 line of NSCLC, which appeared to be more sensitive to the irradiation, which translated to the higher magnitude of senescence at analogical doses of the IR [103].

A dose of 10 Gy was also sufficient to induce senescence in p53 wild-type MCF-7 breast cancer cells [104]. The pro-senescence activity of IR was also confirmed in other p53 wild-type cells, including HCT116 colorectal cancer cells, A172 glioblastoma, and SKNSH neuroblastoma cells. The potent role in the effectory phase of cell cycle inhibition in those cells was played by p21^Cip1^ [105]. There is evidence, however, that the status of p53 matters concerning IR-related inducibility of senescence in cancer cells. Research on MDA-MB231 breast cancer cells having the attenuated p53 function showed that they failed to senesce in response to IR, ending their existence via apoptosis [104].

As for the cells prone to IR-induced senescence, radiation did not suppress the expression of telomerase subunits, change telomerase activity, or induce telomere shortening, suggesting that this kind of the therapy-induced senescence proceeds without telomere attrition, but with evident telomere dysfunction (end-to-end fusions)  [104]. Telomere shortening-independent manner of senescence was also documented in irradiated, SA-β-Gal-positive lung cancer cells [106].

It has recently been found that the choice between apoptosis and senescence in cancer cells exposed to IR may be determined by the status of securin, the multifunctional protein involved in DNA replication, repair [107], and tumorigenesis [108]. It has been found that securin wild-type colon cancer cells subjected to the IR undergo apoptosis. At the same time, in securin-deprived cells, IR triggers senescence [109]. Further research from the same group showed that senescence triggered by IR in securin-deficient human breast cancer cells, MDA-MB-231, involves ATM/Chk2, p38 MAPK, AMPK, and NF-κB [110, 111]. Senescence of these cells in response to IR also involved the activation of glyceraldehyde-3-phosphate dehydrogenase and lactate dehydrogenase A, two enzymes critical for glycolysis. Significantly, an inhibition of glycolysis using dichloroacetate attenuated the ability of the tested cells to undergo IR-dependent senescence [111]. In lung cancer cells, in turn, IR-induced senescence appeared to be regulated by miR-34a [103]. In glioma cells, the final status of cells subjected to IR (apoptosis vs. senescence) is determined by tumor suppressor, PTEN. Namely, PTEN deficiency favored senescence in the irradiated cells, whereas the PTEN proficiency directed the cells towards apoptosis [112].

### Drug-induced senescence of cancer cells

There is a long and still expanding list of drugs that are capable of inducing senescence in cancer cells. This list includes: aphidicolin, bleomycin, cisplatin, doxorubicin, etoposide, mitoxantrone, retinols, hydroxyurea, carboplatin combined with docetaxel, and many others [101]. Majority of these agents act via the induction of DNA damage, albeit they also include inhibitors of DNA polymerase, reactive oxygen species generating agents, and differentiation agents. The pro-senescence activity of chemotherapeutics was revealed in multiple tumors, including breast, lung, prostate, and colon cancer [101], irrespective of p53 status [113]. On the other hand, drugs characterized by different modes of action differ in their capacity to induce senescence. A comparative analysis employing equitoxic concentrations of drugs showed that the strongest pro-senescence response was found in the case of DNA-damaging agents, whereas the weakest effect was observed in the case of drugs targeting microtubules [114].

Senescence of cancer cells in response to chemotherapy is one possible fate experienced by these cells, next to necrosis or apoptosis. The outcome is plausibly linked with the magnitude of a stressor applied: the stronger insult causes cell death, whereas the weaker stimulus leads to senescence. Such a dichotomy was observed, e.g., in prostate cancer cells which underwent apoptotic cell death upon the treatment with 250 nM of doxorubicin [115], or cellular senescence when the drug concentration was 10 times lower [116]. In general, however, the drug-induced senescence of cancer cells proceeds at a lower dynamic than in pro-apoptotic reactions. The expression of SA-β-Gal, and characteristic hypertrophic morphology of cancer cells, usually takes at least few days (3–7) after the treatment [101]. It should also be stressed that senescence is initiated at lower doses than those causing cell death, which may minimalize plausible side effects of the therapy [101].

Interestingly, cancer cells unable to undergo apoptosis, including those lacking p53 and pRb, maintain their propensity to senesce with concomitant sensitivity to chemotherapeutics [117]. There are also situations when the activation of senescence is mandatory for effective therapy. This was observed, e.g., in murine lymphoma in which an intact senescence response determined the effectiveness of cyclophosphamide [117]. A similar effect was found in the case of arsenic trioxide–retinoic acid therapy in acute promyelocytic leukemia [118].

Several of the drugs that are capable of inducing senescence in cancer cells act via the destruction of DNA, mainly by causing single- and double-strand breaks [119]. This feature implies that mechanisms of drug-induced senescence of cancer cells and SIPS, or even replicative senescence of normal somatic cells, may have the same core. As for the role of telomeres, the drug-induced senescence seems to proceed by the telomere-independent mechanism, as breast cancer cells exposed to doxorubicin did not display telomere shortening, albeit the accumulation of some cytogenetic changes within these structures was found [120].

At the level of the cell cycle, the effectory phase of senescence involves the same spectrum of inhibitory proteins as in case of somatic cells, which may seem to some extent odd, as several tumors are deprived of functional p16^INK4a^, p21^Cip1^, and p53. This is the case, e.g., for colon cancer cells treated with doxorubicin in which senescence-like growth arrest occurred, either in wild-type cells or in cells with a homozygous knockout of p53 or p21^Cip1^ (though the magnitude of the process was lower) [121]. Another report showed that as much as 20% of tumors in which cellular senescence was elicited in response to chemotherapy displayed mutated p53 [122]. At the same time, there are reports in which p53-dependent senescence was activated [118]. Another example of this is the senescence of p53 wild-type MCF-7 breast cancer cells exposed to doxorubicin [120]. The induction of p21^Cip1^, which may serve as senescence promoter independently from the signals from p53, was observed in colon cancer cells subjected to 6-anilino-5,8-quinolinequinone [123]. In renal carcinoma cells treated with sunitinib, senescence approached, in turn, in a mechanism involving p53, but without a contribution of p21^Cip1^ [124].

As for p16^INK4a^, osteosarcomas engineered to be deficient in this inhibitor were characterized by impaired senescence [125]. The prominent role of p16^INK4a^ was also demonstrated in cisplatin-resistant NSCLC cells which became much more sensitive to low doses of the drug with concomitant senescence upon their transfection with a construct encoding the complete sequence of p16^INK4a^ [126].

It should also be mentioned that senescence of tumor cells lacking functional cell cycle inhibitors may be explained by defects in senescence-associated ribosome biogenesis and concomitant accumulation of rRNA precursors and ribosomal proteins. From the mechanistic point of view, senescent cells accumulate the ribosomal protein S14 (RPS14 or uS11) in the soluble non-ribosomal compartment, where it binds and inhibits cyclin-dependent kinase 4, leading to the inhibition of retinoblastoma protein phosphorylation, cell cycle arrest, and senescence [127].

Despite the similarity between patterns of senescence in normal and cancer cells at the level of the cell cycle, there are also some differences. Probably the most evident is a broader spectrum of phases at which the replication may be arrested, contrasting cancer cells with their somatic partners in which senescence-associated growth arrest occurs mainly in G_1_ or G_2_ phases of the cell cycle [36]. LS174T colon cancer cells treated moderate doses of DNA topoisomerase I inhibitor, SN-38, were growth-arrested in late S and G_2_-M phases, whereas cells subjected to high concentrations of the drug were growth-arrested in G_1_ phase [122]. The G_1_ phase was also a point at which ovarian cancer cells treated with poly(ADP-ribose) polymerase inhibitor, olaparib, became growth-arrested with concomitant expression of SA-β-Gal and the presence of SAHF [128]. In some cases, however, cancer cells have an ability to bypass the checkpoints controlling the cell cycle progression, which eventually leads to endoreplication of their DNA [129].

### Clinical aspects of the therapy-induced senescence

Apart from artificial in vitro conditions, the therapy-induced senescence of cancer cells has repeatedly been evidenced in tumors in vivo. This is the case, for example, for breast tumors from patients who had received neoadjuvant chemotherapy, in which about 40% of tumors displayed a positive reaction for SA–β–Gal and p16^INK4a^ [122]. The same was documented in NSCLC patients subjected to carboplatin and paclitaxel [130].

The presence of senescent cancer cells in response to chemotherapy in vivo was also found in various animal models, including breast carcinoma exposed to retinoid [113], lymphoma treated with cyclophosphamide [117], and lung NSCLC treated with cisplatin [131].

**Fig. 2:**
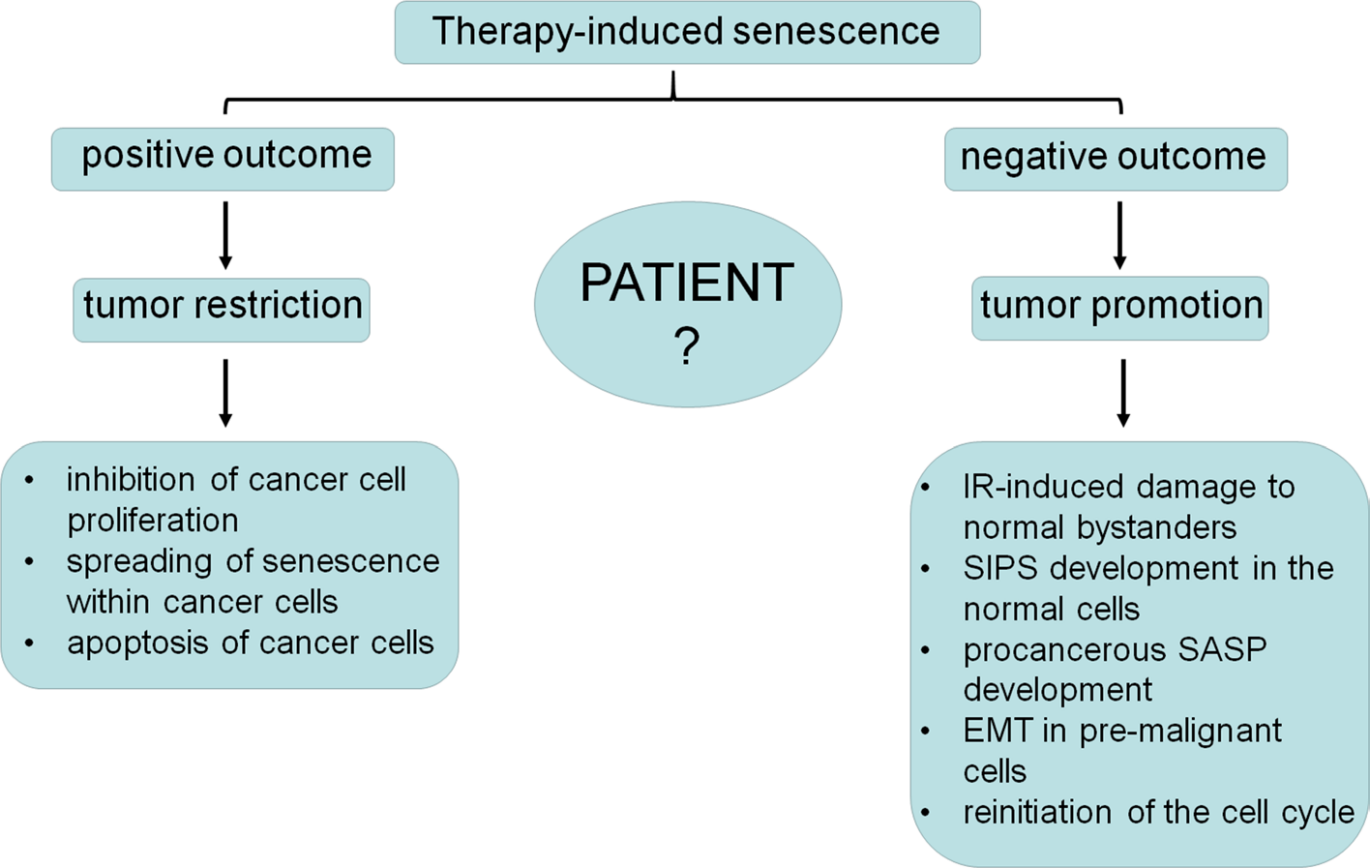
Therapy-induced senescence of cancer cells from the perspective of the ambivalent, either positive or negative outcomes of this process for a patient

These facts indicate that the therapy-induced senescence should be considered from two perspectives: positive and negative for a patient (Fig. 2). The positive aspect of the senescence relies on the growth inhibition of targeted cells, which, at least theoretically, restricts the progression of the disease. Another positive effect is the spreading of senescence towards neighboring cancer cells, as shown in case of MCF-7 breast cancer cells, which, upon exposure to conditioned medium from their senescent counterparts became growth-arrested, enlarged and positive for SA-β-Gal. This is, however, not a universal process, as HCT-116 colon cancer cells did not display signs of senescence in response to conditioned medium from senescent cells [132].

At the same time, potentially adverse effects of the therapy-induced senescence seem to match, and maybe even prevail the pros of this phenomenon. It is well known that IR-dependent damage often occurs (especially at low doses of the radiation) not directly in the irradiated cells but in cells which are in close or distant communication with them (so-called radiation-induced non-targeted bystander; NTE). IR-dependent damage generates DNA damage and multiple cellular and molecular alterations [133], which may lead, at least theoretically, to SIPS, and the development of cancer-promoting reactions. As for chemotherapy, cancer cells exposed to DNA-damaging chemotherapy display the same SASP characteristics as normal cells. This trait translated, in pre-malignant epithelial cells, to the development of EMT and the improvement of their invasiveness, mainly in IL-6- and CXCL8-dependent mechanisms [134].

Moreover, chemotherapeutics known to cause senescence in cancer cells may also induce this event in normal, stromal cells. For instance, cisplatin has been found to induce senescence in fibroblasts [135], whereas paclitaxel in vascular endothelial cells [136]. Taking into account that senescent fibroblasts are one of the plausible sources of cancer-associated fibroblasts (CAFs) [137], the acquisition of a senescent phenotype by these cells may lead to paradoxical exacerbation of the disease. This threat was confirmed in studies using transgenic mice that permit tracking and eliminating senescent cells. This study showed that the elimination of doxorubicin-treated senescent fibroblasts reduced side effects of the therapy, including systemic inflammation, bone marrow suppression and heart failure [138]. Tumors may also benefit from senescence-inducing treatments directly, due to the inactivation of critical oncogenes and/or restoration of tumor-inhibitory signals which may permit cancerous cells to adequately respond to intrinsic damage [139]. This was found in cancer cells associated with the interventions directed towards overexpressed c-Myc overexpression [125] and Shp2 [140], and insufficient PTEN [141].

Another challenge is the stability of cancer cell senescence, which in case of normal somatic cells is considered irreversible [142]. It was observed that a small fraction of H1299 NSCLC cells that undergone senescence in response to genotoxins escaped from senescence and reentered the cell cycle. Interestingly, both the phenotype and gene expression profile of these cells were more similar to senescent cells than to parental cells [130]. A similar phenomenon was demonstrated in HCT116 colon cancer cells. It is very likely that such the reinitiation of divisions stems from the polyploidization of senescent cancer [129].

Taking all above-mentioned facts into account, therapy-induced senescence cannot be treated merely as an alternative therapeutic solution for oncologic patients. There seems to be, however, a chance to take such an approach seriously when the senescence-inducing therapy could be restricted exclusively to cancerous cells or reasonably combined with specific, e.g., SASP-targeting senolytics [143], alternatively drugs which block the SASP without eliminating senescent cells, referred to as senostatics [144]. Recent years provided multiple information regarding biological effects exerted by the both classes of anti-senescence drugs, that allow to consider them as valuable adjuvants in cancer therapy. More precisely, they should be treated as secondary therapy aimed at eliminating therapy-driven senescent cells. Such the activity has been evidenced, e.g., for ABT263, a compound that kills senescent cells generated by chemotherapy, by targeting BCL-2-dependent anti-apoptotic signaling [145]. In addition, in vivo experiments in mouse models revealed that ABT263-dependent clearance of therapy-induced senescent cells translated to reduced cancer metastasis and relapse [138]. Other report showed that the administration of ABT263 enhances the efficiency of clinically relevant therapeutic regimens [146]. As per senostatics, their potential to effectively synergize with chemotherapeutics appears to be even higher. Probably the best examples for this activity derive from experiments on dietary restriction [147] and anti-diabetic drug, metformin [148].

## Spontaneous senescence of cancer cells

To render to Caesar what is Caesar’s, the presence of senescent cancer cells in tumors from patients who had not received any form of radio- or chemotherapy was described several years ago; however, it may have been underestimated and considered a background for much more extensive therapy-induced senescence [122].

### The occurrence of spontaneously senescent cancer cells

It has been observed that 10% of tumors isolated from patients suffering from breast cancer, who had not received chemotherapy, expressed SA-β-Gal. The pattern of staining in these sections differed from that observed in tumors from patients subjected to chemotherapy. In the non-treated tumors, only individual cells were SA-β-Gal-positive, whereas treated tumors revealed patches of cells expressing the SA-β-Gal [122]. Another example of the in vivo detection of senescent cells derives from studies on xenografts generated in athymic nude mice in which H-*ras*-transformed MCF10ANeoT cells formed hyperplastic and pre-malignant tumors characterized by ductal hyperplasia and carcinoma in situ [149]. Analysis of frozen tumor sections from untreated mice showed detectable expression of SA-β-Gal, which was localized to limited tumor fragments and significantly lower compared with the enzyme staining in tumors from animals treated with 4-hydroxyphenyl retinamide [113]. An expression of SA-β-Gal combined with the presence of other markers of senescence, including p16^Ink4a^ and p21^Cip1^ cell cycle inhibitors has also been observed in Reed–Sternberg cells within Hodgkin’s lymphoma biopsies [150].

The phenomenon of spontaneous senescence of cancer cells was also observed in vitro in case of MDA-MB-231 breast cancer cells. Although these cells have active telomerase, which prevents telomere shortening and allows cells to proliferate indefinitely, there was a fraction of cells that lost the ability to divide. These cells have been marked according to the positive expression of SA-β-Gal and negative tritiated thymidine index, in a cellular proliferation test, combined with an enlarged morphology, and their fraction reached 2.2%. In addition, 19.6% of cells were SA-β-Gal-positive but incorporated the thymidine, so their senescence profile was incomplete. Analysis of telomeres showed that proliferating and senescent cells had comparable telomere lengths, suggesting that spontaneous senescence of these cells is telomere-independent, similar to cells undergoing therapy-induced growth arrest [6]. The size of the spontaneously senescent cell fraction confirmed a report by another group, which revealed that 1–3% of untreated sub-confluent fibrosarcoma cells express SA-β-Gal. Noteworthy, the percentage of these cells was remarkably lower compared with cultures treated with doxorubicin [113].

Somehow different light on the nature of the spontaneous senescence of cancer cells sheds reports in which the phenomenon is analyzed in cultured primary prostate, breast, and colon cancer cells, as well as in established MCF7 and MDA-MB-468 breast cancer cells, SW962 vulvar cancer, melanoma SK-MEL28 cells, and lung cancer cells NCI-H1975 and NCI-H460. Authors of this study showed that all primary cancer cells tested undergo the spontaneous senescence, and that a small fraction of senescent cells has also been observed in four of six established cancer cell lines [151]. As for the phenotype of senescent cancer cells, all cultures exhibiting this trait were positive for SA-β-Gal, flattened and elongated morphology, but at the same time, none of them displayed another classic sign of senescence, that is, the presence of SAHF [151]. Significantly, the fraction of senescent cells increased during consecutive passages leading to the stage at which almost the whole population consisted of the senescent cells. The pace at which this complete senescence was achieved was, however, variable, even within cultures of the same origin. The last observation is not strange in the context of the knowledge about replicative lifespan of somatic cells, where two cells derived from a single mitosis may display considerably different numbers of achievable divisions, due to several stochastic events experienced during culture in vitro [152].

What should be stressed at this point is the fact that, conversely to small percentages of spontaneously senescent cells claimed in previously cited papers, the size of the fraction in the tested established and primary cultures ranged from less than 10% in the former to 80–90% in the latter. These last values are, in fact, relatively high which may indicate that the cells passed through several divisions and became senescent during the establishment of the primary culture. There is also the possibility that the cancer cells used in the discussed paper are particularly vulnerable to environmental conditions, such as the ambient oxygen tension, in which they may resemble some types of normal somatic cells which have a substantial fraction of cells bearing various markers of senescence even at very early passages [153]. Such a phenomenon is often called sudden senescence syndrome [154].

The stochastic pattern of the growth and spontaneous senescence was also reported in hepatocellular carcinoma-derived Huh7 cells [155]. Some of the clones established from this line were able to reach more than 100 population doublings with a heterogenous pattern of SA-β-Gal staining, whereas other clones entered senescence much more rapidly and the majority of cells were SA-β-Gal-positive. Similar to normal somatic cells, senescent Huh7 cells remained viable for several months [156] and did not produce immortal progeny.

Diversified dynamics of senescence occurrence was also demonstrated in primary cultures established from malignant ascites collected from ovarian cancer patients. Upon the confirmation of cancerous nature of these cells, the analysis of their behavior and phenotype showed that late-passage cells (usually starting from passages 4–5 onwards) begin to exhibit alterations in morphology and reduced capacity to replicate. The rate of senescence was, however, different, in some cultures senescence occurred at the second passage, and in some at the eighth passage [157]. Considerably later onset of senescence was observed in p53-positive glioblastoma cells. In that case, the spontaneous presence of senescent cells [SA-β-Gal(+)/BrdU(−)] was recorded between the fifteenth and twentieth passage [158].

### Plausible mechanisms of spontaneous cancer cell senescence

As for plausible mechanisms of spontaneous cancer cell senescence, the data are obscure. Research cited above show that the occurrence of spontaneous senescence in primary and stable cancer cell lines may be not related to p53 status, since it was found in both p53 wild-type and mutated cells [151, 158, 159]. In p53- and p16-deficient Huh7 hepatocellular cancer cells the spontaneous senescence is accompanied by the repression of hTERT and telomere shortening [155]. Mechanistically, this process was associated with the activity of the *SIP1* gene, coding for zinc-finger homeodomain transcription factor protein involved in TGF-β signaling [160]. It has been found that targeting of *SIP1* with shRNA restored the hTERT and allowed the cells to bypass senescence [155]. The sensitivity for the induction of senescence has been found to be related to the interplay between oncogenic transcription factor E2F1 and oncoprotein CIP2A. The positive interaction between these two molecules is initiated by the inactivation of p53, which eventually leads to the inhibition of senescence in breast cancer cells [161].

There are reports showing that the spontaneous senescence of cancer cells may be linked to the activity of some oncogenes (OIS). A spontaneous occurrence of OIS has been described in melanocytes, in particular, in their pre-malignant form, melanocytic nevi, that frequently bear activating mutations in *BRAF* and *NRAS* [162]. In breast cancer cells, OIS is triggered by an overexpression of human pituitary tumor-transforming gene 1 *(hPTTG1*) [163].

The role of oncogenes also confirms in vivo observations on mouse having a conditional oncogenic K-*ras* V12 allele. Upon the activation of the oncogene with Cre recombinase, the animals develop multiple pre-malignant lung adenomas and a few lung adenocarcinomas. Research using cryosections of lungs from K-*ras* V12 animals showed strong expression of SA-β-Gal in the pre-malignant lesions, which coincided with low expression of Ki67-proliferative antigen, and significant staining of p16^INK4a^, p15, Dec1, and DcR2, markers of senescence identified previously using microarray analysis on cell cultures. At the same time, it should be emphasized that authors of the study did not reveal the presence of senescence in the malignant tumors, which may indicate their resistance to OIS, likely due to the loss of effectory proteins, p16^INK4a^ and p53 [164].

In sections of atypical proliferative serous ovarian tumors, which are precursors of low-grade serous ovarian cancer, the presence of cells positive for p16^INK4a^ and with low Ki67 labeling index—considered as being senescent—was found in a fraction of cells with abundant eosinophilic cytoplasm, bearing mutated *BRAF* proto-oncogene. This interpretation supported further in vitro analyses using epithelial cells with ectopic expression of BRAF^V600E^, which expressed such the hallmarks of senescence, as SA-β-Gal and high expression of p16^INK4a^ and p21^Cip1^ [165].

OIS has also been revealed as a barrier in the development of lymphoma. It has been demonstrated that primary lymphocytes engineered to express *RAS* became SA-β-Gal-positive, which terminated lymphomagenesis at its very initial step. This *RAS*-induced senescence was also strongly related to the activity of histone methyltransferase, Suv39h1, involved in the methylation of histone H3 lysine 9 (H3 K9me), being a part of the retinoblastoma signaling [166].

On the other hand, the rationale regarding the involvement of the OIS in the spontaneous cancer cells senescence is challenged by the observation of the lack of SAHF in senescent primary and established cell lines [151]. Indeed, it has been postulated that the formation of SAHF is a unique feature of OIS [167], which in the face of the above-cited study could indicate that mechanisms outside of OIS may underlie the spontaneous induction of this phenomenon.

An OIS-independent scenario may include the loss of phosphatase and tensin homolog deleted on chromosome 10 (*PTEN*), one of the most frequently mutated tumor suppressor genes in prostate cancer [168]. Seminal experiments using mouse embryonic fibroblasts (MEFs) showed that cells deprived of *PTEN* exhibit senescence-like morphology and SA-β-Gal staining. This effect was associated with the activation of the PI3K/AKT signaling and proceeded in a clearly p53-dependent manner. Notably, strong SA-β-Gal staining was also found in *PTEN*-deficient, pre-neoplastic prostates in mice. In these lesions, the accumulated p53 was accompanied by an increase in p19 and p21^Cip1^. A strong SA-β-Gal reaction was also found in early-stage human prostate cancer. In the areas of full-blown carcinoma, the staining was still present, but its magnitude was weaker [169]. The role of *PTEN* inhibition as a cause of cancer cell senescence strengthened further in vivo research on p53 wild type MDA PCa-2b xenograft prostate tumors, in which chemical targeting of *PTEN* resulted in an increased expression of SA-β-Gal and decreased expression of Ki67 [170]. Significantly, the induction of senescence related to the inhibition of *PTEN* does not involve initial hyperproliferation and DNA damage response, which may be more profitable from the perspective of therapeutic application compared with OIS, in which both these reactions are present [171].

**Fig. 3:**
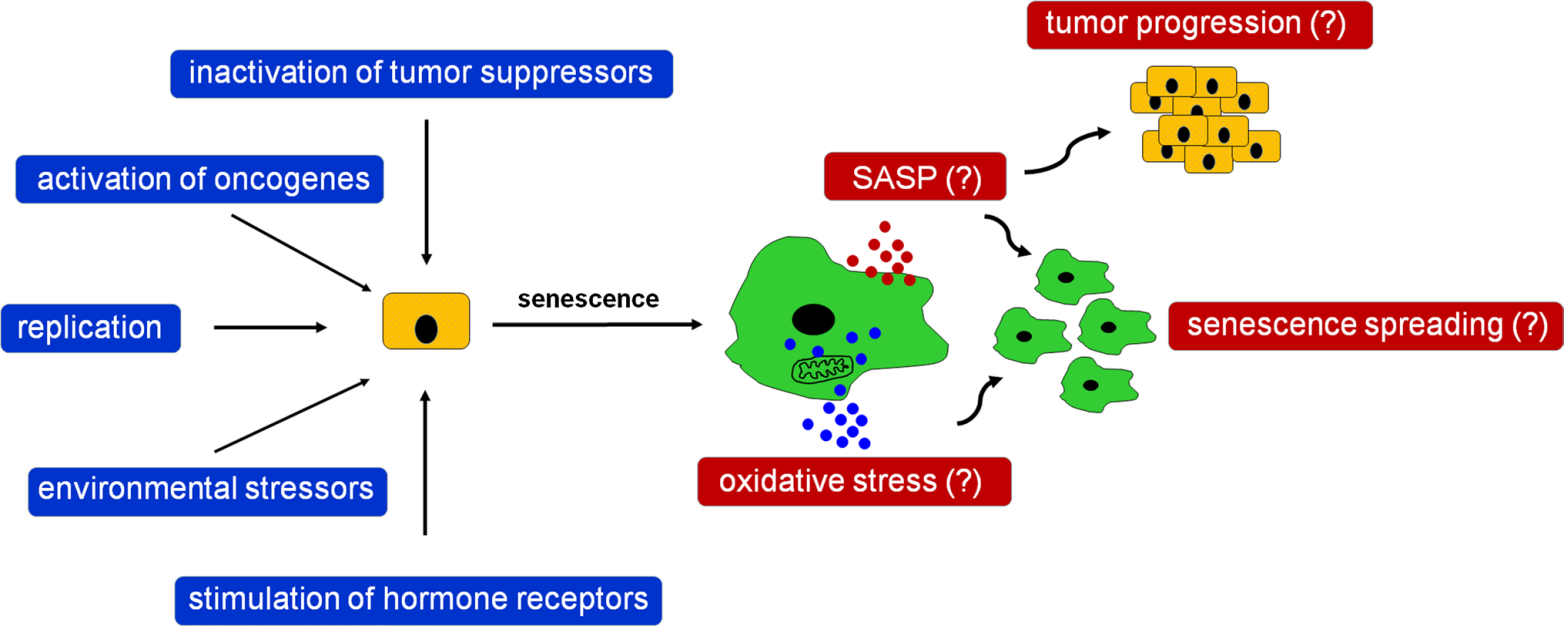
Theoretical triggers of spontaneous senescence of cancer cells in the context of plausible biological activities of these cells. According to the literature, spontaneous senescence of cancer cells may be a complex phenomenon, elicited in vivo by various kinds of stressors of both exogenous and endogenous nature. Spontaneously senescent cancer cells display similar phenotype to normal senescent cells and cancer cells undergoing IR or chemotherapy, including the development of SASP and increased oxidative stress. The SASP may further lead to the exacerbation of cancer progression, but at the same time, its elements can also be responsible for spreading of senescence among nearby proliferating cells, simultaneously with pro-senescence effects generated by oxidative stress

Another mechanism of spontaneous senescence induction has been proposed according to research on ovarian cancer cells. Researchers showed that ES-2 cells expressing progesterone receptor (PR), PEO4 cells positive for the PR and estrogen receptors, and primary ovarian cancer cells, displayed various features of senescence, including growth arrest in the G_1_ phase of cell cycle, an enlarged morphology, an expression of SA-β-Gal, and upregulated levels of p21^Cip1^ upon the experimental stimulation of the PR. Interestingly, although the p21^Cip1^ cell cycle inhibitor seemed initially to be the core effector of this response, inhibition of p21^Cip1^ with shRNA paradoxically increased SA-β-Gal, with a concomitant upregulation—as a compensatory response, as speculate authors of the study—of other cell cycle inhibitors, such as p15, p16^INK4a^, and p27. Notably, the most critical molecule controlling senescence in the PR-positive ovarian cancer cells appeared to be transcription factor FOXO1, whose stable inhibition effectively prevented all pro-senescence effects of the PR stimulation [172]. Plausible triggers and potential biological outcomes of spontaneous cancer cell senescence have been summarized in Fig. 3.

## Conclusions and further remarks

Taking into account the uncertain clinical outcomes of the drug-induced senescence of cancer cells, one may ask as to whether even a small fraction of spontaneously senescent cancer cells should not attract some attention regarding its possible biological and clinical relevance. In our opinion, the answer is yes, due to the analysis of the literature focused on a similar phenomenon in various tumor types showing that spontaneous senescence is a general rule rather than a negligible artifact. Unfortunately, the phenomenological description of the presence of spontaneously senescent cancer cells in vivo and in vitro has not yet been appropriately followed by mechanistic studies. This seriously jeopardizes our knowledge about the molecular nature of this process. Also, in terms of the comparative analysis with the therapy-induced counterpart, we firmly believe that there is an urgent need to experimentally identify the molecular nature of these cells and possible results of cell interactions with proliferating, non-senescent counterparts and stromal cells. One of the most critical issues to be addressed is of whether the relatively small fraction of spontaneously senescent cells is biologically active, and if so, whether this activity resembles the activity of cells that senesced in response to chemotherapy or radiation. Or, whether the presence and activity of spontaneously senescent cells may jeopardize cancer cell response to chemotherapy. It should also be clarified if biological effects of spontaneously senescent cells and the cells forced to senescence by therapy are synergistic or maybe opposed (due to different dynamics, magnitudes, and perhaps also mechanisms?). Last but not least, further investigations using cell line and in vivo models should be performed to delineate whether the fraction of spontaneously senescent cells increases over time, strengthening the understanding of their potential activity.

## References

[1] Carrel A (1912). On the permanent life of tissues outside of the organism. J Exp Med.

[2] Hayflick L, Moorhead PS (1961). The serial cultivation of human diploid cell strains. Exp Cell Res.

[3] Hayflick L (1965). The limited in vitro lifetime of human diploid cell strains. Exp Cell Res.

[4] Shay JW, Zou Y, Hiyama E, Wright WE (2001). Telomerase and cancer. Hum Mol Genet.

[5] Roninson IB (2003). Tumor cell senescence in cancer treatment. Cancer Res.

[6] Cukusic A, Ivankovic M, Skrobot N, Ferenac M, Gotic I, Matijasic M, Polancec D, Rubelj I (2006). Spontaneous senescence in the MDA-MB-231 cell line. Cell Prolif.

[7] Carrel A (1928). The Immortality of Animal Tissues and Its Significance. Can Med Assoc J.

[8] Cristofalo VJ, Volker C, Allen RG (2000). Use of the fibroblast model in the study of cellular senescence. Methods Mol Med.

[9] Ksiazek K, Korybalska K, Jorres A, Witowski J (2007). Accelerated senescence of human peritoneal mesothelial cells exposed to high glucose: the role of TGF-beta1. Lab Invest.

[10] Chondrogianni N, Stratford FL, Trougakos IP, Friguet B, Rivett AJ, Gonos ES (2003). Central role of the proteasome in senescence and survival of human fibroblasts: induction of a senescence-like phenotype upon its inhibition and resistance to stress upon its activation. J Biol Chem.

[11] Krouwer VJ, Hekking LH, Langelaar-Makkinje M, Regan-Klapisz E, Post JA (2012). Endothelial cell senescence is associated with disrupted cell-cell junctions and increased monolayer permeability. Vasc Cell.

[12] Statuto M, Bianchi C, Perego R, Del MU (2002). Drop of connexin 43 in replicative senescence of human fibroblasts HEL-299 as a possible biomarker of senescence. Exp Gerontol.

[13] Nishio K, Inoue A, Qiao S, Kondo H, Mimura A (2001). Senescence and cytoskeleton: overproduction of vimentin induces senescent-like morphology in human fibroblasts. Histochem Cell Biol.

[14] Dikovskaya D, Cole JJ, Mason SM, Nixon C, Karim SA, McGarry L, Clark W, Hewitt RN, Sammons MA, Zhu J, Athineos D, Leach JD, Marchesi F, van Tuyn J, Tait SW, Brock C, Morton JP, Wu H, Berger SL, Blyth K, Adams PD (2015). Mitotic Stress Is an Integral Part of the Oncogene-Induced Senescence Program that Promotes Multinucleation and Cell Cycle Arrest. Cell Rep.

[15] Denoyelle C, Abou-Rjaily G, Bezrookove V, Verhaegen M, Johnson TM, Fullen DR, Pointer JN, Gruber SB, Su LD, Nikiforov MA, Kaufman RJ, Bastian BC, Soengas MS (2006). Anti-oncogenic role of the endoplasmic reticulum differentially activated by mutations in the MAPK pathway. Nat Cell Biol.

[16] Passos JF, Nelson G, Wang C, Richter T, Simillion C, Proctor CJ, Miwa S, Olijslagers S, Hallinan J, Wipat A, Saretzki G, Rudolph KL, Kirkwood TB, von Zglinicki T (2010). Feedback between p21 and reactive oxygen production is necessary for cell senescence. Mol Syst Biol.

[17] Wiley CD, Velarde MC, Lecot P, Liu S, Sarnoski EA, Freund A, Shirakawa K, Lim HW, Davis SS, Ramanathan A, Gerencser AA, Verdin E, Campisi J (2016). Mitochondrial dysfunction induces senescence with a distinct secretory phenotype. Cell Metab.

[18] Ksiazek K, Piatek K, Witowski J (2008). Impaired response to oxidative stress in senescent cells may lead to accumulation of DNA damage in mesothelial cells from aged donors. Biochem Biophys Res Commun.

[19] Rodier F, Munoz DP, Teachenor R, Chu V, Le O, Bhaumik D, Coppe JP, Campeau E, Beausejour CM, Kim SH, Davalos AR, Campisi J (2011). DNA-SCARS: distinct nuclear structures that sustain damage-induced senescence growth arrest and inflammatory cytokine secretion. J Cell Sci.

[20] Aird KM, Zhang R (2013). Detection of senescence-associated heterochromatin foci (SAHF). Methods Mol Biol.

[21] Sidler C, Kovalchuk O, Kovalchuk I (2017). Epigenetic regulation of Cellular senescence and aging. Front Genet.

[22] Dimri GP, Lee X, Basile G, Acosta M, Scott G, Roskelley C, Medrano EE, Linskens M, Rubelj I, Pereira-Smith O, Campisi J (1995). A biomarker that identifies senescent human cells in culture and in aging skin in vivo. Proc Natl Acad Sci U S A.

[23] Severino J, Allen RG, Balin S, Balin A, Cristofalo VJ (2000). Is beta-galactosidase staining a marker of senescence in vitro and in vivo?. Exp Cell Res.

[24] Cristofalo VJ (2005). SA beta gal staining: biomarker or delusion. Exp Gerontol.

[25] Coppe JP, Desprez PY, Krtolica A, Campisi J (2010). The senescence-associated secretory phenotype: the dark side of tumor suppression. Annu Rev Pathol.

[26] Calio A, Zamo A, Ponzoni M, Zanolin ME, Ferreri AJ, Pedron S, Montagna L, Parolini C, Fraifeld VE, Wolfson M, Yanai H, Pizzolo G, Doglioni C, Vinante F, Chilosi M (2015). Cellular senescence markers p16INK4a and p21CIP1/WAF are predictors of hodgkin lymphoma outcome. Clin Cancer Res.

[27] Evangelou K, Lougiakis N, Rizou SV, Kotsinas A, Kletsas D, Munoz-Espin D, Kastrinakis NG, Pouli N, Marakos P, Townsend P, Serrano M, Bartek J, Gorgoulis VG (2017). Robust, universal biomarker assay to detect senescent cells in biological specimens. Aging Cell.

[28] Lawless C, Wang C, Jurk D, Merz A, Zglinicki T, Passos JF (2010). Quantitative assessment of markers for cell senescence. Exp Gerontol.

[29] Galbiati A, Beausejour C, d’Adda di Fagagna F (2017). A novel single-cell method provides direct evidence of persistent DNA damage in senescent cells and aged mammalian tissues. Aging Cell.

[30] von Zglinicki T, Saretzki G, Ladhoff J, d`Adda di Fagagna F, Jackson SP (2005). Human cell senescence as a DNA damage response. Mech Ageing Dev.

[31] Bielak-Zmijewska A, Mosieniak G, Sikora E (2018). Is DNA damage indispensable for stress-induced senescence?. Mech Ageing Dev.

[32] Kural KC, Tandon N, Skoblov M, Kel-Margoulis OV, Baranova AV (2016). Pathways of aging: comparative analysis of gene signatures in replicative senescence and stress induced premature senescence. BMC Genomics.

[33] Jia P, Her C, Chai W (2015). DNA excision repair at telomeres. DNA Repair (Amst).

[34] d`Adda di Fagagna F, Reaper PM, Clay-Farrace L, Fiegler H, Carr P, von Zglinicki T, Saretzki G, Carter NP, Jackson SP (2003). A DNA damage checkpoint response in telomere-initiated senescence. Nature.

[35] Marechal A, Zou L (2013). DNA damage sensing by the ATM and ATR kinases. Cold Spring Harb Perspect Biol.

[36] Gire V, Dulic V (2015). Senescence from G2 arrest, revisited. Cell Cycle.

[37] Qian Y, Chen X (2013). Senescence regulation by the p53 protein family. Methods Mol Biol.

[38] d’Adda di Fagagna F (2008). Living on a break: cellular senescence as a DNA-damage response. Nat Rev Cancer.

[39] Fumagalli M, Rossiello F, Mondello C, d’Adda di Fagagna F (2014). Stable cellular senescence is associated with persistent DDR activation. PLoS One.

[40] Ksiazek K, Breborowicz A, Jorres A, Witowski J (2007). Oxidative stress contributes to accelerated development of the senescent phenotype in human peritoneal mesothelial cells exposed to high glucose. Free Radic Biol Med.

[41] Kang MK, Guo W, Park NH (1998). Replicative senescence of normal human oral keratinocytes is associated with the loss of telomerase activity without shortening of telomeres. Cell Growth Differ.

[42] Sherr CJ, DePinho RA (2000). Cellular senescence: mitotic clock or culture shock?. Cell.

[43] Ksiazek K, Mikula-Pietrasik J, Olijslagers S, Jorres A, von Zglinicki T, Witowski J (2009). Vulnerability to oxidative stress and different patterns of senescence in human peritoneal mesothelial cell strains. Am J Physiol Regul Integr Comp Physiol.

[44] Ksiazek K, Passos JF, Olijslagers S, Saretzki G, Martin-Ruiz C, von Zglinicki T (2007). Premature senescence of mesothelial cells is associated with non-telomeric DNA damage. Biochem Biophys Res Commun.

[45] Ramirez RD, Morales CP, Herbert BS, Rohde JM, Passons C, Shay JW, Wright WE (2001). Putative telomere-independent mechanisms of replicative aging reflect inadequate growth conditions. Genes Dev.

[46] Alcorta DA, Xiong Y, Phelps D, Hannon G, Beach D, Barrett JC (1996). Involvement of the cyclin-dependent kinase inhibitor p16 (INK4a) in replicative senescence of normal human fibroblasts. Proc Natl Acad Sci USA.

[47] Moon KC, Yang JP, Lee JS, Jeong SH, Dhong ES, Han SK (2018). Effects of ultraviolet irradiation on cellular senescence in keratinocytes versus fibroblasts. J Craniofac Surg.

[48] Wang Y, Boerma M, Zhou D (2016). Ionizing radiation-induced endothelial cell senescence and cardiovascular diseases. Radiat Res.

[49] Zhao W, Lin ZX, Zhang ZQ (2004). Cisplatin-induced premature senescence with concomitant reduction of gap junctions in human fibroblasts. Cell Res.

[50] Zdanov S, Remacle J, Toussaint O (2006). Establishment of H2O2-induced premature senescence in human fibroblasts concomitant with increased cellular production of H2O2. Ann N Y Acad Sci.

[51] Duan J, Duan J, Zhang Z, Tong T (2005). Irreversible cellular senescence induced by prolonged exposure to H2O2 involves DNA-damage-and-repair genes and telomere shortening. Int J Biochem Cell Biol.

[52] Chen QM, Prowse KR, Tu VC, Purdom S, Linskens MH (2001). Uncoupling the senescent phenotype from telomere shortening in hydrogen peroxide-treated fibroblasts. Exp Cell Res.

[53] Dimauro T, David G (2010). Ras-induced senescence and its physiological relevance in cancer. Curr Cancer Drug Targets.

[54] Jeanblanc M, Ragu S, Gey C, Contrepois K, Courbeyrette R, Thuret JY, Mann C (2012). Parallel pathways in RAF-induced senescence and conditions for its reversion. Oncogene.

[55] Chandeck C, Mooi WJ (2010). Oncogene-induced cellular senescence. Adv Anat Pathol.

[56] Passos JF, Saretzki G, Ahmed S, Nelson G, Richter T, Peters H, Wappler I, Birket MJ, Harold G, Schaeuble K, Birch-Machin MA, Kirkwood TB, von Zglinicki T (2007). Mitochondrial dysfunction accounts for the stochastic heterogeneity in telomere-dependent senescence. PLoS Biol.

[57] von Zglinicki T, Saretzki G, Docke W, Lotze C (1995). Mild hyperoxia shortens telomeres and inhibits proliferation of fibroblasts: a model for senescence?. Exp Cell Res.

[58] Baird DM (2008). Telomere dynamics in human cells. Biochimie.

[59] Ksiazek K, Passos JF, Olijslagers S, von Zglinicki T (2008). Mitochondrial dysfunction is a possible cause of accelerated senescence of mesothelial cells exposed to high glucose. Biochem Biophys Res Commun.

[60] Allen RG, Tresini M, Keogh BP, Doggett DL, Cristofalo VJ (1999). Differences in electron transport potential, antioxidant defenses, and oxidant generation in young and senescent fetal lung fibroblasts (WI-38). J Cell Physiol.

[61] Venkatachalam G, Surana U, Clement MV (2017). Replication stress-induced endogenous DNA damage drives cellular senescence induced by a sub-lethal oxidative stress. Nucleic Acids Res.

[62] Poulios E, Trougakos IP, Chondrogianni N, Gonos ES (2007). Exposure of human diploid fibroblasts to hypoxia extends proliferative life span. Ann N Y Acad Sci.

[63] Tsai CC, Chen YJ, Yew TL, Chen LL, Wang JY, Chiu CH, Hung SC (2011). Hypoxia inhibits senescence and maintains mesenchymal stem cell properties through down-regulation of E2A-p21 by HIF-TWIST. Blood.

[64] Gorissen B, de Bruin A, Miranda-Bedate A, Korthagen N, Wolschrijn C, de Vries TJ, van Weeren R, Tryfonidou MA (2018). Hypoxia negatively affects senescence in osteoclasts and delays osteoclastogenesis. J Cell Physiol.

[65] Lee SH, Lee JH, Yoo SY, Hur J, Kim HS, Kwon SM (2013). Hypoxia inhibits cellular senescence to restore the therapeutic potential of old human endothelial progenitor cells via the hypoxia-inducible factor-1alpha-TWIST-p21 axis. Arterioscler Thromb Vasc Biol.

[66] Kilic Eren M, Tabor V (2014). The role of hypoxia inducible factor-1 alpha in bypassing oncogene-induced senescence. PLoS One.

[67] Beausejour CM, Krtolica A, Galimi F, Narita M, Lowe SW, Yaswen P, Campisi J (2003). Reversal of human cellular senescence: roles of the p53 and p16 pathways. EMBO J.

[68] Park JK, Kim BH, Han YS, Park IK (2002). The effect of telomerase expression on the escape from M2 crisis in virus-transformed human retinal pigment epithelial cells. Exp Mol Med.

[69] Rubin H (1997). Cell aging in vivo and in vitro. Mech Ageing Dev.

[70] Verzola D, Gandolfo MT, Gaetani G, Ferraris A, Mangerini R, Ferrario F, Villaggio B, Gianiorio F, Tosetti F, Weiss U, Traverso P, Mji M, Deferrari G, Garibotto G (2008). Accelerated senescence in the kidneys of patients with type 2 diabetic nephropathy. Am J Physiol Renal Physiol.

[71] Childs BG, Baker DJ, Wijshake T, Conover CA, Campisi J, van Deursen JM (2016). Senescent intimal foam cells are deleterious at all stages of atherosclerosis. Science.

[72] Castro P, Giri D, Lamb D, Ittmann M (2003). Cellular senescence in the pathogenesis of benign prostatic hyperplasia. Prostate.

[73] Sosinska P, Mikula-Pietrasik J, Ryzek M, Naumowicz E, Ksiazek K (2014). Specificity of cytochemical and fluorescence methods of senescence-associated beta-galactosidase detection for ageing driven by replication and time. Biogerontology.

[74] Baker DJ, Childs BG, Durik M, Wijers ME, Sieben CJ, Zhong J, Saltness RA, Jeganathan KB, Verzosa GC, Pezeshki A, Khazaie K, Miller JD, van Deursen JM (2016). Naturally occurring p16(Ink4a)-positive cells shorten healthy lifespan. Nature.

[75] Palmer AK, Tchkonia T, LeBrasseur NK, Chini EN, Xu M, Kirkland JL (2015). Cellular senescence in Type 2 diabetes: a therapeutic opportunity. Diabetes.

[76] Martin JA, Brown TD, Heiner AD, Buckwalter JA (2004). Chondrocyte senescence, joint loading and osteoarthritis. Clin Orthop Relat Res.

[77] Fu Q, Qin Z, Yu J, Yu Y, Tang Q, Lyu D, Zhang L, Chen Z, Yao K (2016). Effects of senescent lens epithelial cells on the severity of age-related cortical cataract in humans: a case-control study. Medicine (Baltimore).

[78] Michaloglou C, Vredeveld LC, Soengas MS, Denoyelle C, Kuilman T, van der Horst CM, Majoor DM, Shay JW, Mooi WJ, Peeper DS (2005). BRAFE600-associated senescence-like cell cycle arrest of human naevi. Nature.

[79] Anderson R, Richardson GD, Passos JF (2018). Mechanisms driving the ageing heart. Exp Gerontol.

[80] Bussian TJ, Aziz A, Meyer CF, Swenson BL, van Deursen JM, Baker DJ (2018). Clearance of senescent glial cells prevents tau-dependent pathology and cognitive decline. Nature.

[81] Mikula-Pietrasik J, Uruski P, Sosinska P, Maksin K, Piotrowska-Kempisty H, Kucinska M, Murias M, Szubert S, Wozniak A, Szpurek D, Sajdak S, Piwocka K, Tykarski A, Ksiazek K (2016). Senescent peritoneal mesothelium creates a niche for ovarian cancer metastases. Cell Death Dis.

[82] Demaria M, Ohtani N, Youssef SA, Rodier F, Toussaint W, Mitchell JR, Laberge RM, Vijg J, Van Steeg H, Dolle ME, Hoeijmakers JH, de Bruin A, Hara E, Campisi J (2014). An essential role for senescent cells in optimal wound healing through secretion of PDGF-AA. Dev Cell.

[83] Storer M, Mas A, Robert-Moreno A, Pecoraro M, Ortells MC, Di Giacomo V, Yosef R, Pilpel N, Krizhanovsky V, Sharpe J, Keyes WM (2013). Senescence is a developmental mechanism that contributes to embryonic growth and patterning. Cell.

[84] Vicente R, Mausset-Bonnefont AL, Jorgensen C, Louis-Plence P, Brondello JM (2016). Cellular senescence impact on immune cell fate and function. Aging Cell.

[85] Ritschka B, Storer M, Mas A, Heinzmann F, Ortells MC, Morton JP, Sansom OJ, Zender L, Keyes WM (2017). The senescence-associated secretory phenotype induces cellular plasticity and tissue regeneration. Genes Dev.

[86] Zhu Y, Tchkonia T, Pirtskhalava T, Gower AC, Ding H, Giorgadze N, Palmer AK, Ikeno Y, Hubbard GB, Lenburg M, O’Hara SP, LaRusso NF, Miller JD, Roos CM, Verzosa GC, LeBrasseur NK, Wren JD, Farr JN, Khosla S, Stout MB, McGowan SJ, Fuhrmann-Stroissnigg H, Gurkar AU, Zhao J, Colangelo D, Dorronsoro A, Ling YY, Barghouthy AS, Navarro DC, Sano T, Robbins PD, Niedernhofer LJ, Kirkland JL (2015). The Achilles’ heel of senescent cells: from transcriptome to senolytic drugs. Aging Cell.

[87] Campisi J (2005). Senescent cells, tumor suppression, and organismal aging: good citizens, bad neighbors. Cell.

[88] Ruhland MK, Loza AJ, Capietto AH, Luo X, Knolhoff BL, Flanagan KC, Belt BA, Alspach E, Leahy K, Luo J, Schaffer A, Edwards JR, Longmore G, Faccio R, DeNardo DG, Stewart SA (2016). Stromal senescence establishes an immunosuppressive microenvironment that drives tumorigenesis. Nat Commun.

[89] Hernandez-Segura A, de Jong TV, de Jong TV, Melov S, Guryev V, Campisi J, Demaria M (2017). Unmasking Transcriptional Heterogeneity in Senescent Cells. Curr Biol.

[90] Coppe JP, Kauser K, Campisi J, Beausejour CM (2006). Secretion of vascular endothelial growth factor by primary human fibroblasts at senescence. J Biol Chem.

[91] Taddei ML, Cavallini L, Comito G, Giannoni E, Folini M, Marini A, Gandellini P, Morandi A, Pintus G, Raspollini MR, Zaffaroni N, Chiarugi P (2014). Senescent stroma promotes prostate cancer progression: the role of miR-210. Mol Oncol.

[92] Borodkina AV, Deryabin PI, Giukova AA, Nikolsky NN (2018). “Social Life” of senescent cells: what is SASP and why study it?. Acta Naturae.

[93] Ito Y, Hoare M, Narita M (2017). Spatial and temporal control of senescence. Trends Cell Biol.

[94] Rodier F, Coppe JP, Patil CK, Hoeijmakers WA, Munoz DP, Raza SR, Freund A, Campeau E, Davalos AR, Campisi J (2009). Persistent DNA damage signalling triggers senescence-associated inflammatory cytokine secretion. Nat Cell Biol.

[95] Freund A, Patil CK, Campisi J (2011). p38MAPK is a novel DNA damage response-independent regulator of the senescence-associated secretory phenotype. EMBO J.

[96] Liu D, Hornsby PJ (2007). Senescent human fibroblasts increase the early growth of xenograft tumors via matrix metalloproteinase secretion. Cancer Res.

[97] Mikula-Pietrasik J, Sosinska P, Maksin K, Kucinska MG, Piotrowska H, Murias M, Wozniak A, Szpurek D, Ksiazek K (2015). Colorectal cancer-promoting activity of the senescent peritoneal mesothelium. Oncotarget.

[98] Wang T, Notta F, Navab R, Joseph J, Ibrahimov E, Xu J, Zhu CQ, Borgida A, Gallinger S, Tsao MS (2017). Senescent carcinoma-associated fibroblasts upregulate IL8 to enhance prometastatic phenotypes. Mol Cancer Res.

[99] Mikula-Pietrasik J, Sosinska P, Naumowicz E, Maksin K, Piotrowska H, Wozniak A, Szpurek D, Ksiazek K (2016). Senescent peritoneal mesothelium induces a pro-angiogenic phenotype in ovarian cancer cells in vitro and in a mouse xenograft model in vivo. Clin Exp Metastasis.

[100] Ivancich M, Schrank Z, Wojdyla L, Leviskas B, Kuckovic A, Sanjali A, Puri N (2017). Treating cancer by targeting telomeres and telomerase. Antioxidants (Basel).

[101] Ewald JA, Desotelle JA, Wilding G, Jarrard DF (2010). Therapy-induced senescence in cancer. J Natl Cancer Inst.

[102] Lomax ME, Folkes LK, O’Neill P (2013). Biological consequences of radiation-induced DNA damage: relevance to radiotherapy. Clin Oncol (R Coll Radiol).

[103] He X, Yang A, McDonald DG, Riemer EC, Vanek KN, Schulte BA, Wang GY (2017). MiR-34a modulates ionizing radiation-induced senescence in lung cancer cells. Oncotarget.

[104] Jones KR, Elmore LW, Jackson-Cook C, Demasters G, Povirk LF, Holt SE, Gewirtz DA (2005). p53-Dependent accelerated senescence induced by ionizing radiation in breast tumour cells. Int J Radiat Biol.

[105] Mirzayans R, Scott A, Cameron M, Murray D (2005). Induction of accelerated senescence by gamma radiation in human solid tumor-derived cell lines expressing wild-type TP53. Radiat Res.

[106] Suzuki K, Mori I, Nakayama Y, Miyakoda M, Kodama S, Watanabe M (2001). Radiation-induced senescence-like growth arrest requires TP53 function but not telomere shortening. Radiat Res.

[107] Jallepalli PV, Waizenegger IC, Bunz F, Langer S, Speicher MR, Peters JM, Kinzler KW, Vogelstein B, Lengauer C (2001). Securin is required for chromosomal stability in human cells. Cell.

[108] Tfelt-Hansen J, Kanuparthi D, Chattopadhyay N (2006). The emerging role of pituitary tumor transforming gene in tumorigenesis. Clin Med Res.

[109] Chen WS, Yu YC, Lee YJ, Chen JH, Hsu HY, Chiu SJ (2010). Depletion of securin induces senescence after irradiation and enhances radiosensitivity in human cancer cells regardless of functional p53 expression. Int J Radiat Oncol Biol Phys.

[110] Yu YC, Yang PM, Chuah QY, Huang YH, Peng CW, Lee YJ, Chiu SJ (2013). Radiation-induced senescence in securin-deficient cancer cells promotes cell invasion involving the IL-6/STAT3 and PDGF-BB/PDGFR pathways. Sci Rep.

[111] Liao EC, Hsu YT, Chuah QY, Lee YJ, Hu JY, Huang TC, Yang PM, Chiu SJ (2014). Radiation induces senescence and a bystander effect through metabolic alterations. Cell Death Dis.

[112] Lee JJ, Kim BC, Park MJ, Lee YS, Kim YN, Lee BL, Lee JS (2011). PTEN status switches cell fate between premature senescence and apoptosis in glioma exposed to ionizing radiation. Cell Death Differ.

[113] Chang BD, Broude EV, Dokmanovic M, Zhu H, Ruth A, Xuan Y, Kandel ES, Lausch E, Christov K, Roninson IB (1999). A senescence-like phenotype distinguishes tumor cells that undergo terminal proliferation arrest after exposure to anticancer agents. Cancer Res.

[114] Vergel M, Marin JJ, Estevez P, Carnero A (2010). Cellular senescence as a target in cancer control. J Aging Res.

[115] Ewald JA, Peters N, Desotelle JA, Hoffmann FM, Jarrard DF (2009). A high-throughput method to identify novel senescence-inducing compounds. J Biomol Screen.

[116] Schwarze SR, Fu VX, Desotelle JA, Kenowski ML, Jarrard DF (2005). The identification of senescence-specific genes during the induction of senescence in prostate cancer cells. Neoplasia.

[117] Schmitt CA, Fridman JS, Yang M, Lee S, Baranov E, Hoffman RM, Lowe SW (2002). A senescence program controlled by p53 and p16INK4a contributes to the outcome of cancer therapy. Cell.

[118] Ablain J, Rice K, Soilihi H, de Reynies A, Minucci S, de The H (2014). Activation of a promyelocytic leukemia-tumor protein 53 axis underlies acute promyelocytic leukemia cure. Nat Med.

[119] Cheung-Ong K, Giaever G, Nislow C (2013). DNA-damaging agents in cancer chemotherapy: serendipity and chemical biology. Chem Biol.

[120] Elmore LW, Rehder CW, Di X, McChesney PA, Jackson-Cook CK, Gewirtz DA, Holt SE (2002). Adriamycin-induced senescence in breast tumor cells involves functional p53 and telomere dysfunction. J Biol Chem.

[121] Chang BD, Xuan Y, Broude EV, Zhu H, Schott B, Fang J, Roninson IB (1999). Role of p53 and p21waf1/cip1 in senescence-like terminal proliferation arrest induced in human tumor cells by chemotherapeutic drugs. Oncogene.

[122] te Poele RH, Okorokov AL, Jardine L, Cummings J, Joel SP (2002). DNA damage is able to induce senescence in tumor cells in vitro and in vivo. Cancer Res.

[123] Lodygin D, Menssen A, Hermeking H (2002). Induction of the Cdk inhibitor p21 by LY83583 inhibits tumor cell proliferation in a p53-independent manner. J Clin Invest.

[124] Zhu Y, Xu L, Zhang J, Hu X, Liu Y, Yin H, Lv T, Zhang H, Liu L, An H, Liu H, Xu J, Lin Z (2013). Sunitinib induces cellular senescence via p53/Dec1 activation in renal cell carcinoma cells. Cancer Sci.

[125] Wu CH, van Riggelen J, Yetil A, Fan AC, Bachireddy P, Felsher DW (2007). Cellular senescence is an important mechanism of tumor regression upon c-Myc inactivation. Proc Natl Acad Sci USA.

[126] Fang K, Chiu CC, Li CH, Chang YT, Hwang HT (2007). Cisplatin-induced senescence and growth inhibition in human non-small cell lung cancer cells with ectopic transfer of p16INK4a. Oncol Res.

[127] Lessard F, Igelmann S, Trahan C, Huot G, Saint-Germain E, Mignacca L, Del Toro N, Lopes-Paciencia S, Le Calve B, Montero M, Deschenes-Simard X, Bury M, Moiseeva O, Rowell MC, Zorca CE, Zenklusen D, Brakier-Gingras L, Bourdeau V, Oeffinger M, Ferbeyre G (2018). Senescence-associated ribosome biogenesis defects contributes to cell cycle arrest through the Rb pathway. Nat Cell Biol.

[128] Wang ZG, Zhou J, Liu H, Xu C (2019). Olaparib induced senescence under p16 or p53 dependent manner in ovarian cancer. J Gynecol Oncol.

[129] Sliwinska MA, Mosieniak G, Wolanin K, Babik A, Piwocka K, Magalska A, Szczepanowska J, Fronk J, Sikora E (2009). Induction of senescence with doxorubicin leads to increased genomic instability of HCT116 cells. Mech Ageing Dev.

[130] Roberson RS, Kussick SJ, Vallieres E, Chen SY, Wu DY (2005). Escape from therapy-induced accelerated cellular senescence in p53-null lung cancer cells and in human lung cancers. Cancer Res.

[131] Ge H, Ni S, Wang X, Xu N, Liu Y, Wang X, Wang L, Song D, Song Y, Bai C (2012). Dexamethasone reduces sensitivity to cisplatin by blunting p53-dependent cellular senescence in non-small cell lung cancer. PLoS One.

[132] Di X, Bright AT, Bellott R, Gaskins E, Robert J, Holt S, Gewirtz D, Elmore L (2008). A chemotherapy-associated senescence bystander effect in breast cancer cells. Cancer Biol Ther.

[133] Yahyapour R, Salajegheh A, Safari A, Amini P, Rezaeyan A, Amraee A, Najafi M (2018). Radiation-induced non-targeted effect and carcinogenesis; implications in clinical radiotherapy. J Biomed Phys Eng.

[134] Coppe JP, Patil CK, Rodier F, Sun Y, Munoz DP, Goldstein J, Nelson PS, Desprez PY, Campisi J (2008). Senescence-associated secretory phenotypes reveal cell-nonautonomous functions of oncogenic RAS and the p53 tumor suppressor. PLoS Biol.

[135] Peiris-Pages M, Sotgia F, Lisanti MP (2015). Chemotherapy induces the cancer-associated fibroblast phenotype, activating paracrine Hedgehog-GLI signalling in breast cancer cells. Oncotarget.

[136] Ota H, Eto M, Ako J, Ogawa S, Iijima K, Akishita M, Ouchi Y (2009). Sirolimus and everolimus induce endothelial cellular senescence via sirtuin 1 down-regulation: therapeutic implication of cilostazol after drug-eluting stent implantation. J Am Coll Cardiol.

[137] Alspach E, Fu Y, Stewart SA (2013). Senescence and the pro-tumorigenic stroma. Crit Rev Oncog.

[138] Demaria M, O’Leary MN, Chang J, Shao L, Liu S, Alimirah F, Koenig K, Le C, Mitin N, Deal AM, Alston S, Academia EC, Kilmarx S, Valdovinos A, Wang B, de Bruin A, Kennedy BK, Melov S, Zhou D, Sharpless NE, Muss H, Campisi J (2017). Cellular senescence promotes adverse effects of chemotherapy and cancer relapse. Cancer Discov.

[139] Childs BG, Durik M, Baker DJ, van Deursen JM (2015). Cellular senescence in aging and age-related disease: from mechanisms to therapy. Nat Med.

[140] Serrano M (2015). SHP2: a new target for pro-senescence cancer therapies. EMBO J.

[141] Toso A, Revandkar A, Di Mitri D, Guccini I, Proietti M, Sarti M, Pinton S, Zhang J, Kalathur M, Civenni G, Jarrossay D, Montani E, Marini C, Garcia-Escudero R, Scanziani E, Grassi F, Pandolfi PP, Catapano CV, Alimonti A (2014). Enhancing chemotherapy efficacy in Pten-deficient prostate tumors by activating the senescence-associated antitumor immunity. Cell Rep.

[142] Takahashi A, Ohtani N, Hara E (2007). Irreversibility of cellular senescence: dual roles of p16INK4a/Rb-pathway in cell cycle control. Cell Div.

[143] Birch J, Passos JF (2017). Targeting the SASP to combat ageing: mitochondria as possible intracellular allies?. Bioessays.

[144] Short S, Fielder E, Miwa S, von Zglinicki T (2019). Senolytics and senostatics as adjuvant tumour therapy. EBioMedicine.

[145] Wang L, Leite de Oliveira R, Wang C, Fernandes Neto JM, Mainardi S, Evers B, Lieftink C, Morris B, Jochems F, Willemsen L, Beijersbergen RL, Bernards R (2017). High-Throughput Functional Genetic and Compound Screens Identify Targets for Senescence Induction in Cancer. Cell Rep.

[146] Tse C, Shoemaker AR, Adickes J, Anderson MG, Chen J, Jin S, Johnson EF, Marsh KC, Mitten MJ, Nimmer P, Roberts L, Tahir SK, Xiao Y, Yang X, Zhang H, Fesik S, Rosenberg SH, Elmore SW (2008). ABT-263: a potent and orally bioavailable Bcl-2 family inhibitor. Cancer Res.

[147] Harvey AE, Lashinger LM, Hays D, Harrison LM, Lewis K, Fischer SM, Hursting SD (2014). Calorie restriction decreases murine and human pancreatic tumor cell growth, nuclear factor-kappaB activation, and inflammation-related gene expression in an insulin-like growth factor-1-dependent manner. PLoS One.

[148] Campbell JM, Bellman SM, Stephenson MD, Lisy K (2017). Metformin reduces all-cause mortality and diseases of ageing independent of its effect on diabetes control: a systematic review and meta-analysis. Ageing Res Rev.

[149] Miller FR, Soule HD, Tait L, Pauley RJ, Wolman SR, Dawson PJ, Heppner GH (1993). Xenograft model of progressive human proliferative breast disease. J Natl Cancer Inst.

[150] Gopas J, Stern E, Zurgil U, Ozer J, Ben-Ari A, Shubinsky G, Braiman A, Sinay R, Ezratty J, Dronov V, Balachandran S, Benharroch D, Livneh E (2016). Reed-Sternberg cells in Hodgkin’s lymphoma present features of cellular senescence. Cell Death Dis.

[151] Zieba J, Ksiazkiewcz M, Janik K, Banaszczyk M, Peciak J, Piaskowski S, Lipinski M, Olczak M, Stoczynska-Fidelus E, Rieske P (2015). Sensitivity of neoplastic cells to senescence unveiled under standard cell culture conditions. Anticancer Res.

[152] Smith JR, Whitney RG (1980). Intraclonal variation in proliferative potential of human diploid fibroblasts: stochastic mechanism for cellular aging. Science.

[153] Ksiazek K, Mikula-Pietrasik J, Jorres A, Witowski J (2008). Oxidative stress-mediated early senescence contributes to the short replicative life span of human peritoneal mesothelial cells. Free Radic Biol Med.

[154] Rubelj I, Huzak M, Brdar B, Pereira-Smith OM (2002). A single-stage mechanism controls replicative senescence through Sudden Senescence Syndrome. Biogerontology.

[155] Ozturk N, Erdal E, Mumcuoglu M, Akcali KC, Yalcin O, Senturk S, Arslan-Ergul A, Gur B, Yulug I, Cetin-Atalay R, Yakicier C, Yagci T, Tez M, Ozturk M (2006). Reprogramming of replicative senescence in hepatocellular carcinoma-derived cells. Proc Natl Acad Sci USA.

[156] De Cecco M, Criscione SW, Peckham EJ, Hillenmeyer S, Hamm EA, Manivannan J, Peterson AL, Kreiling JA, Neretti N, Sedivy JM (2013). Genomes of replicatively senescent cells undergo global epigenetic changes leading to gene silencing and activation of transposable elements. Aging Cell.

[157] Rl OD, McCormick A, Mukhopadhyay A, Woodhouse LC, Moat M, Grundy A, Dixon M, Kaufman A, Soohoo S, Elattar A, Curtin NJ, Edmondson RJ (2014). The use of ovarian cancer cells from patients undergoing surgery to generate primary cultures capable of undergoing functional analysis. PLoS One.

[158] Stoczynska-Fidelus E, Piaskowski S, Bienkowski M, Banaszczyk M, Hulas-Bigoszewska K, Winiecka-Klimek M, Radomiak-Zaluska A, Och W, Borowiec M, Zieba J, Treda C, Rieske P (2014). The failure in the stabilization of glioblastoma-derived cell lines: spontaneous in vitro senescence as the main culprit. PLoS One.

[159] Stoczynska-Fidelus E, Och W, Rieske P, Bienkowski M, Banaszczyk M, Winiecka-Klimek M, Zieba J, Janik K, Rosiak K, Treda C, Stawski R, Radomiak-Zaluska A, Piaskowski S (2014). Spontaneous in vitro senescence of glioma cells confirmed by an antibody against IDH1R132H. Anticancer Res.

[160] Verschueren K, Remacle JE, Collart C, Kraft H, Baker BS, Tylzanowski P, Nelles L, Wuytens G, Su MT, Bodmer R, Smith JC, Huylebroeck D (1999). SIP1, a novel zinc finger/homeodomain repressor, interacts with Smad proteins and binds to 5′-CACCT sequences in candidate target genes. J Biol Chem.

[161] Laine A, Sihto H, Come C, Rosenfeldt MT, Zwolinska A, Niemela M, Khanna A, Chan EK, Kahari VM, Kellokumpu-Lehtinen PL, Sansom OJ, Evan GI, Junttila MR, Ryan KM, Marine JC, Joensuu H, Westermarck J (2013). Senescence sensitivity of breast cancer cells is defined by positive feedback loop between CIP2A and E2F1. Cancer Discov.

[162] Bansal R, Nikiforov MA (2010). Pathways of oncogene-induced senescence in human melanocytic cells. Cell Cycle.

[163] Ruan JW, Liao YC, Lua I, Li MH, Hsu CY, Chen JH (2012). Human pituitary tumor-transforming gene 1 overexpression reinforces oncogene-induced senescence through CXCR163/p21 signaling in breast cancer cells. Breast Cancer Res.

[164] Collado M, Gil J, Efeyan A, Guerra C, Schuhmacher AJ, Barradas M, Benguria A, Zaballos A, Flores JM, Barbacid M, Beach D, Serrano M (2005). Tumour biology: senescence in premalignant tumours. Nature.

[165] Zeppernick F, Ardighieri L, Hannibal CG, Vang R, Junge J, Kjaer SK, Zhang R, Kurman RJ, Shih I (2014). BRAF mutation is associated with a specific cell type with features suggestive of senescence in ovarian serous borderline (atypical proliferative) tumors. Am J Surg Pathol.

[166] Braig M, Lee S, Loddenkemper C, Rudolph C, Peters AH, Schlegelberger B, Stein H, Dorken B, Jenuwein T, Schmitt CA (2005). Oncogene-induced senescence as an initial barrier in lymphoma development. Nature.

[167] Kosar M, Bartkova J, Hubackova S, Hodny Z, Lukas J, Bartek J (2011). Senescence-associated heterochromatin foci are dispensable for cellular senescence, occur in a cell type- and insult-dependent manner and follow expression of p16(ink4a). Cell Cycle.

[168] Li J, Yen C, Liaw D, Podsypanina K, Bose S, Wang SI, Puc J, Miliaresis C, Rodgers L, McCombie R, Bigner SH, Giovanella BC, Ittmann M, Tycko B, Hibshoosh H, Wigler MH, Parsons R (1997). PTEN, a putative protein tyrosine phosphatase gene mutated in human brain, breast, and prostate cancer. Science.

[169] Chen Z, Trotman LC, Shaffer D, Lin HK, Dotan ZA, Niki M, Koutcher JA, Scher HI, Ludwig T, Gerald W, Cordon-Cardo C, Pandolfi PP (2005). Crucial role of p53-dependent cellular senescence in suppression of Pten-deficient tumorigenesis. Nature.

[170] Alimonti A, Nardella C, Chen Z, Clohessy JG, Carracedo A, Trotman LC, Cheng K, Varmeh S, Kozma SC, Thomas G, Rosivatz E, Woscholski R, Cognetti F, Scher HI, Pandolfi PP (2010). A novel type of cellular senescence that can be enhanced in mouse models and human tumor xenografts to suppress prostate tumorigenesis. J Clin Invest.

[171] Di Micco R, Fumagalli M, Cicalese A, Piccinin S, Gasparini P, Luise C, Schurra C, Garre M, Nuciforo PG, Bensimon A, Maestro R, Pelicci PG, d’Adda di Fagagna F (2006). Oncogene-induced senescence is a DNA damage response triggered by DNA hyper-replication. Nature.

[172] Diep CH, Charles NJ, Gilks CB, Kalloger SE, Argenta PA, Lange CA (2013). Progesterone receptors induce FOXO1-dependent senescence in ovarian cancer cells. Cell Cycle.

